# No evidence for changes in skeletal muscle mass or weight during first-line chemotherapy for metastatic colorectal cancer

**DOI:** 10.1186/s12885-019-6086-2

**Published:** 2019-08-28

**Authors:** Sami Antoun, Mohamed Amine Bayar, Valérie Dyevre, Emilie Lanoy, Cristina Smolenschi, Michel Ducreux

**Affiliations:** 1 Medical Emergency Unit in Oncology, Gustave Roussy Cancer Campus, 94800 Villejuif, France; 2 Department of Biostatics and Epidemiology, Gustave Roussy Cancer Campus, 94800 Villejuif, France; 3 CESP, Faculté de Médecine, Université Paris Sud, INSERM, Université Paris Saclay, 94805 Villejuif, France; 4 Department Medical Oncology, Gustave Roussy Cancer Campu, 94800 Villejuif, France

**Keywords:** Muscle mass depletion, Metastatic colorectal cancer, Weight loss, Body composition, Overall survival, Chemotherapy toxicity

## Abstract

**Background:**

Studies over the past 10 years strongly support an association between skeletal muscle mass (SMM) depletion and outcome in metastatic colorectal cancer (mCRC). Factors influencing SMM changes over time are, however, poorly studied. We analyzed the impact of SMM on overall survival and chemotherapy toxicities in mCRC patients treated with first-line chemotherapy. Changes in weight and body composition were evaluated during follow-up.

**Methods:**

Patients enrolled in the randomized phase II ACCORD trial comparing two chemotherapy regimens were screened. Body composition parameters (SMM, adipose tissue) were assessed prospectively with computed tomography (CT) imaging, and toxicities were recorded. Mixed models were used to assess weight and BC changes during 4 months of treatment follow-up.

**Results:**

Among 145 patients included in ACCORD, 76 had available baseline CT scans and were included in the current study. Mean age was 60.6 ± 10.0 years, 50% were women, 82% had colon cancer, and 62% had two or more metastatic sites. At baseline, 49% had lost at least 5% of their initial weight, including 26% who had lost more than 10%; 53% had SMM depletion. In this homogenous cohort, there were no statistically significant associations between SMM depletion and overall survival, progression-free survival or chemotherapy toxicity. There were no decreases in weight or SMM during follow-up. Weight and SMM changes were not influenced by diarrhea either grade 3–4 or any grade (reported in 74% of patients). For patients with weight loss ≥10% at baseline, SMM increased significantly after 4 months of follow-up and after disease stabilization following chemotherapy (*P* = 0.008).

**Conclusions:**

In a homogenous mCRC cohort, SMM depletion was not associated with survival or chemotherapy toxicity. Despite most patient experiencing diarrhea, no changes in weight or SMM were found during 4 months of follow-up. However, hypotheses deriving from our exploratory study have to be tested in further larger sample size studies.

**Trial registration:**

Clinicaltrials.gov NCT00423696 (2011).

**Electronic supplementary material:**

The online version of this article (10.1186/s12885-019-6086-2) contains supplementary material, which is available to authorized users.

## Background

The prognosis of colorectal cancer (CRC), the third most frequent cancer in the Western world has changed dramatically with the advent of treatments combining cytotoxic drugs, targeted therapies, radiotherapy, and surgical resection of metastases [1]. Median survival in patients with metastatic CRC (mCRC) now exceeds 24 months [2, 3]. Several multidimensional prognostic and predictive factors related to tumor responsiveness to therapies, pathological stage, and the patient’s resistance capacity against the disease, are associated with outcome [4]. Among patient-related factors, nutritional parameters can influence outcomes in cancer patients [5].

In the past 10 years, studies have strongly suggested that skeletal muscle mass (SMM) depletion is associated with poorer survival outcomes independent of weight loss (WL) [6]. In CRC, the deleterious effect of SMM depletion on overall survival (OS) has been described for stage I to III disease [7], for patients undergoing adjuvant chemotherapy [8], as well as for short-term post-operative outcomes [9–12]. SMM depletion has also been associated with chemotherapy toxicity and discontinuation of chemotherapy treatments in many cancer types [13–15], and especially for CRC [8, 16–18].

As with most common solid tumors, CRC disease progression is associated with a progressive nutritional decline [5, 19]. Loss of SMM and adipose tissue may be associated with the intense catabolism linked to progressive disease; in CRC, SMM loss depends on tumor stage and tumor evolution [20] and is accelerated at the end of life [10, 21]. The role of intrinsic factors (patient characteristics, nutritional status, and non-tumoral aspects) and treatment exposure, which could be involved in SMM changes over time, has been poorly studied [22, 23].

Most of the described associations between SMM depletion and outcomes, and most studies on weight and body composition (BC) parameter changes have several limitations including heterogeneity in terms of CRC stages, chemotherapy regimens, and treatment combinations (surgery, radiotherapy). In this study, we selected a homogenous population, in which all patients had the same stage of mCRC, were receiving first-line chemotherapy, and were recruited in the context of a randomized clinical trial evaluating two chemotherapy regimens differentiated only by the modality of administration. Our objective was to analyze the impact of SMM and adipose tissue on OS, progressive-free survival (PFS), as well as on chemotherapy toxicities. The secondary objective was to analyze weight and body composition changes in a homogenous cohort of mCRC patients receiving first-line chemotherapy during the first 4 months after treatment initiation as well as the impact of tumor and patient characteristics on these changes.

## Methods

### Patients

Patients who were enrolled in the ACCORD trial between March 2006 and January 2008 at 15 centers in France, were screened. The ACCORD trial was a prospective multicenter, randomized, open-labelled, non-comparative phase II trial [2]. The aim of the ACCORD study was to evaluate the efficacy and safety of bevacizumab in combination with either oral capecitabine plus irinotecan (XELIRI) or 5-fluorouracil /leucovorin plus irinotecan (FOLFIRI) as first-line therapy for mCRC. To be eligible in the current study, patients had to have available computed tomography (CT) scans at baseline, 2 and 4 months, be aged 18–75 years, with unresectable, histologically proven, measurable mCRC.

The ACCORD study was approved by ethics committee of the Kremlin-Bicêtre hospital and all patients provided informed consent. The additional analyses of clinical data and interpretation of body composition from CT images in the current study were approved by the Gustave Roussy independent ethics committees.

### CT image analysis of anthropometry and body composition

According to the original study protocol, physical examination, and routine blood/urine analysis were assessed within 8 days before starting study treatment and were repeated every treatment cycle. Tumor assessments with abdominal CTs or magnetic resonance imaging were performed at baseline and every 8 weeks until progression. The same scanning techniques were used at each assessment. Weight and height were measured at baseline. For the current study, nutritional follow-up (weight and BC parameter changes) were assessed until the fourth month of treatment. Body mass index (BMI) was calculated (i.e., BMI = weight (kg) / height^2^ (m^2^)).

BC parameters were evaluated using the same CT images obtained for tumor assessment. All CT measurements were performed by the same operator who was blinded to patient information, clinical treatment and outcome. Measured variables were lumbar cross-sectional areas (cm^2^) of skeletal muscle mass (SMM), visceral adipose tissue (VAT), and subcutaneous adipose tissue (SAT), as described previously [22, 24]. The third lumbar vertebra (L3) was chosen as the reference point [25, 26]. CT images were analyzed using Slice-O-Matic software V4·3 (Tomovision, Montreal, Canada). These values were normalized for height scale and expressed in cm^2^/m^2^. To evaluate skeletal muscle density (SMD), we measured the mean radiation attenuation of skeletal muscle, which describes the input images read using Slice-O-Matic software. The pixel values of these images displayed in shades of grey as a correlate of muscle density represent the physical properties of the scanned tissue expressed in a numerical form by the mean Hounsfield Unit [27]. SMM depletion was defined according to the SMM index thresholds described by Martin et al. [6] (for women SMM < 41 cm^2^/m^2^, for men SMM < 43 cm^2^/m^2^ if BMI < 25 kg/m^2^ and < 53 cm^2^/m^2^ if BMI > 25 kg/m^2^).

### Endpoints

According to the original study protocol, OS was defined as time from randomization in the ACCORD study to death or last follow-up. Progression was assessed based on investigators’ tumor assessments. PFS was defined as time from randomization to progression or death not related to progression or last follow-up. Chemotherapy toxicity was evaluated using National Cancer Institute Common Terminology Criteria for Adverse Events, v3.0.

### Statistical analysis

Continuous variables were summarized with the mean and the standard deviation, and the difference according to gender was tested using a Student’s t-test or Mann–Whitney U test depending on normality. Categorical variables were summarized with the number of patients and frequency, and the difference according to gender was tested using a Pearson’s chi-squared test or Fisher exact test depending on the expected numbers. The median follow-up was estimated using the reverse Kaplan-Meier method [28]. Survival was estimated using the Kaplan–Meier product limit method. For OS and PFS, estimates of hazard ratios (HRs) associated with BC parameters, Wald Chi-square *P*-values, and 95% confidence intervals (CIs) were derived from Cox proportional hazard models. Effects of each body composition parameter-L3 skeletal muscle score, L3 visceral adipose tissue score, L3 subcutaneous adipose tissue score, L3 total adipose tissue score, Skeletal muscle density (HU) – on overall survival were derived from six separate multivariable model adjusted for age, sex, number of metastatic sites, treatment and BMI. Survival analyses were also done using Lasso penalized regressions as part of sensitivity analyses. Associations between BC parameters and global or specific toxicity occurrence were identified using a chi-squared test.

Mixed models were used to assess changes in BC parameters during the 4 months of follow-up, for patients with measurements at baseline (T0), 2 months (T2) and 4 months (T4). Linear mixed models were fitted for each BC index: weight, SMM, SMD, VAT and SAT. Full models were adjusted for age and tumor localization and included an interaction between gender and visit (T0, T2, T4). In the selected models, age, localization, and the interaction were only retained at level *p* < .20 in univariable model and according backward selection procedure. Gender was forced in mixed models for body composition, systematically. The correlation structure is a continuous AR (1), autocorrelation structure of order 1, with a continuous time covariate. Only one random effect on the subject was included. For each model (weight, SMM, SMD, VAT and SAT), least square means and contrasts were reported with adjusted *P*-values using the Tukey method and the corresponding 95% CI [29]. Weight loss and SMM depletion definitions refer to baseline. Analyses were performed using SAS Software, version 9.4 (SAS Institute, Cary, NC, USA) and given the sample size, they are considered explanatory.

## Results

### General, tumor, treatment and body composition characteristics

**Fig. 1:**
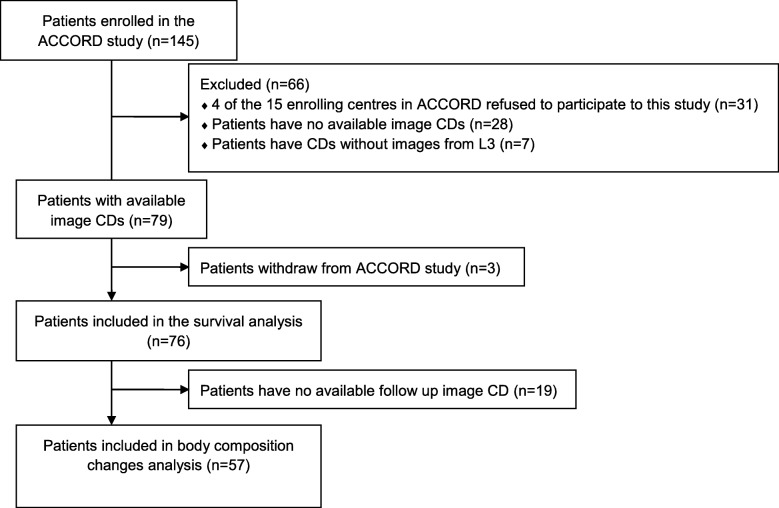
Flow chart for the inclusion process

Among 145 patients included in the ACCORD, 76 met our inclusion criteria and were included in the survival analysis; 69 were excluded (Fig. 1). The mean age (± standard deviation) was 60.6 years (±10.0), and 38 patients (50%) were women (Table 1). Almost all patients (91%) had ECOG PS 0–1, 62% had two or more sites of metastasis and 82% had colon cancer. At baseline only 9% were considered malnourished according to the BMI (< 18.5), and 41% were overweight or obese (BMI > 25). Among 65 patients with available data for their usual weight, 32 (49%) had lost at least 5% of their initial weight at referral including 17 patients (26%) who had lost > 10%. Note that 53% of the population had SMM depletion according to Martin’s thresholds. Fifty-seven patients were finally included in body composition changes analysis.

**Table 1:** Patient, tumor, treatment and body composition characteristics at inclusion

	Male *n* = 38 (50%)	Female *n* = 38 (50%)	Total *n* = 76	*P*-value
Age (years)	60.7 ± 9.1	60.5 ± 11.0	60.6 ± 10.0	0.971
ECOG-PS				1.000
0–1	35 (92%)	34 (90%)	69 (91%)	
2	3 (8%)	4 (10%)	7 (9%)	
Tumor type				1.000
colon	31 (82%)	31 (82%)	62 (82%)	
rectum	7 (18%)	7 (18%)	14 (18%)	
Number of metastatic sites				0.479
1	13 (34%)	16 (42%)	29 (38%)	
≥ 2	25 (66%)	22 (58%)	47 (62%)	
Treatment regimen				0.163
XELIRI	19 (50%)	13 (34%)	32 (42%)	
FOLFIRI	19 (50%)	25 (66%)	44 (58%)	
Weight (kg)	76.4 ± 12.3	57.7 ± 10.8	67.0 ± 14.8	< 0.001
Weight loss (WL) (%)	6.0 ± 6.0	6.0 ± 7.9	6.0 ± 7.0	0.564
WL category 5% cut-off				0.386
≤ 5%	19 (58%)	14 (44%)	33 (51%)	
> 5%	14 (42%)	18 (56%)	32 (49%)	
Missing	5 (−)	6 (−)	11 (−)	
WL category 10% cut-off				0.941
≤ 10%	25 (76%)	23 (72%)	48 (74%)	
> 10%	8 (24%)	9 (28%)	17 (26%)	
Missing	5 (−)	6 (−)	11 (−)	
BMI (kg/m^2^)	25.6 ± 3.8	22.3 ± 4.1	23.9 ± 4.2	< 0.001
BMI category (kg/m^2^)				0.001
< 18.5	0 (0%)	7 (18%)	7 (9%)	
18.5–24.9	16 (42%)	22 (58%)	38 (50%)	
25–29.9	15 (40%)	8 (21%)	23 (30%)	
≥ 30	7 (18%)	1 (3%)	8 (11%)	
SMM index (cm^2^/m^2^)	52.9 ± 9.9	37.9 ± 5.4	45.4 ± 10.9	< 0.001
SMD (HU)	40.4 ± 8.9	40.0 ± 11.2	40.2 ± 10.0	0.881
VAT index (cm^2^/m^2^)	46.5 ± 28.0	23.8 ± 20.5	35.1 ± 26.9	< 0.001
SAT index (cm^2^/m^2^)	45.6 ± 17.5	54.2 ± 30.5	50.0 ± 25.2	0.285
SMM depletion	12 (32%)	28 (74%)	40 (53%)	< 0.001

Continuous variables are summarized with mean ± standard deviation and the difference according to gender is tested using Wilcoxon–Mann–Whitney test. Categorical variables were summarized with the number of patients and the frequency, n (%), and the difference according to gender was tested using a Pearson’s chi-squared test or Fisher exact test when necessary

*ECOG PS* Eastern Cooperative Oncology Group Performance Status, XELIRI bevacizumab + oral capecitabine + irinotecan, *FOLFIRI* bevacizumab + 5-fluorouracil/leucovorin (5FU/LV) + irinotecan

Weight loss (WL) = (usual weight-weight at inclusion) / usual weight × 100; usual weight was available for 65 patients. *BMI* Body Mass Index (weight (kg) / height^2^ (m^2^)), *SMM* skeletal muscle mass, *SMD* skeletal muscle density, *HU* Hounsfield Units, *VAT* visceral adipose tissue, *SAT* subcutaneous adipose tissue

SMM loss thresholds were those defined by Martin et al. [6]

### Overall survival and progression-free survival

For the 76 patients included, the median follow-up (Q1; Q3) was 21 months (16; 29). At the cut-off date, 32 patients died and the median OS was 22 months (95% CI: 20 – NR). In univariate analysis, OS was associated with age (HR = 1.04; 95% CI: 1.00–1.08; *P* = 0.034), and SMM (HR = 0.65; 95% CI: 0.45–0.94 *P* = 0.023). In multivariable analysis, OS was not associated with changes in any BC parameters (SMM depletion, SMM score, VAT score, SAT score, TAT score or skeletal muscle density) (Table 2).

**Table 2:** Univariate and multivariable analyses for overall survival

Characteristic			Univariate analysis		Multivariable^a^ analysis	
	No. evt/ No. pts	Median OS (95% CI)	HR [95% CI]	*P*-value	HR [95% CI]	*P*-value
Age (years)			1.04 [1.00; 1.08]	0.034		
Sex						
Male	17/37	22 (20; NR)	1	0.900		
Female	15/38	21 (20; NR	1.05 [0.52; 2.1]			
ECOG Performance status						
0–1	32/68	21 (20; 24)	NA	NA		
2	0/7	NA	NA			
Number of metastatic sites						
1	11/28	22 (20; NA)	1	0.500		
≥ 2	21/47	22 (19; NA)	1.29 [0.62; 2.68]			
Treatment						
XELIRI	11/31	22 (20; NA)	1	0.150		
FOLFIRI	21/44	21 (20; 24)	1.71 [0.82; 3.56]			
Body mass index category (kg/m^2^)						
< 25	22/45	21 (19; NA)	1	0.180		
≥ 25	10/30	24 (21; NA)	0.60 [0.28; 1.26]			
Model 1: SMM depletion at baseline						
No	13/35	24 (20; NA)	1	0.150	1	0.791
Yes	19/40	21 (20; 23)	1.69 [0.83; 3.43]		1.14 [0.44; 2.98]	
Model 2: L3 skeletal muscle score			0.65 [0.45; 0.94]	0.023	0.71 [0.41; 1.22]	0.211
Model 3: L3 visceral adipose tissue score			1.03 [0.71; 1.48]	0.880	1.05 [0.67; 1.64]	0.833
Model 4: L3 subcutaneous adipose tissue score			0.68 [0.46; 1.01]	0.054	0.71 [0.44; 1.14]	0.153
Model 5: L3 total adipose tissue score			0.84 [0.57; 1.23]	0.370	0.87 [0.53; 1.38]	0.557
Model 6: Skeletal muscle density (HU)			0.98 [0.94; 1.01]	0.210	1.01 [0.95; 1.06]	0.868

*No. evt* number events, *No. pts*. number patients, *HR* Hazard ratio, *CI* confidence interval, *p*-value, *ECOG* Eastern Cooperative Oncology Group, *HU* Hounsfield Units, *NA* not available, *SMM* Skeletal Muscle mass depletion thresholds were those defined by Martin et al. [6]

^a^Each 6 multivariable models assessing effect of each body component parameter on overall survival were adjusted for age, sex, number of metastases, treatment, BMI

A total of 63 patients progressed and the median PFS was 9 months (95% CI: 9–11). In univariate analysis, PFS was associated with age (HR = 1.03; 95% CI: 1.00–1.06; *P* = 0.020), and with SMD (HR = 0.97; 95% CI: 0.95–1.00]; *P* = 0.048). Note that PFS was not associated with any BC parameters and the association with SMD was no longer significant (HR = 1.00; 95% CI: 0.96–1.0,4 *P* = 0.97) (Additional file 1: Table S1).

### Chemotherapy toxicity

Grade 3–4 chemotherapy-related toxicities occurred in 47 (62%) patients. During the first 4 months of treatment, grade 3–4 neutropenia and diarrhea were observed in 22% and 14% of the patients respectively and any grade diarrhea were observed in 74% of the patients. There was no difference between patients with SMM depletion and those without SMM depletion in terms of incidence of any of the chemotherapy toxicities or for toxicities requiring changes to the regimen, irrespective of relationship to XELIRI, FOLFIRI or bevacizumab (Additional file 1: Table S2).

### Changes in body composition parameters and body weight during follow-up

**Fig. 2:**
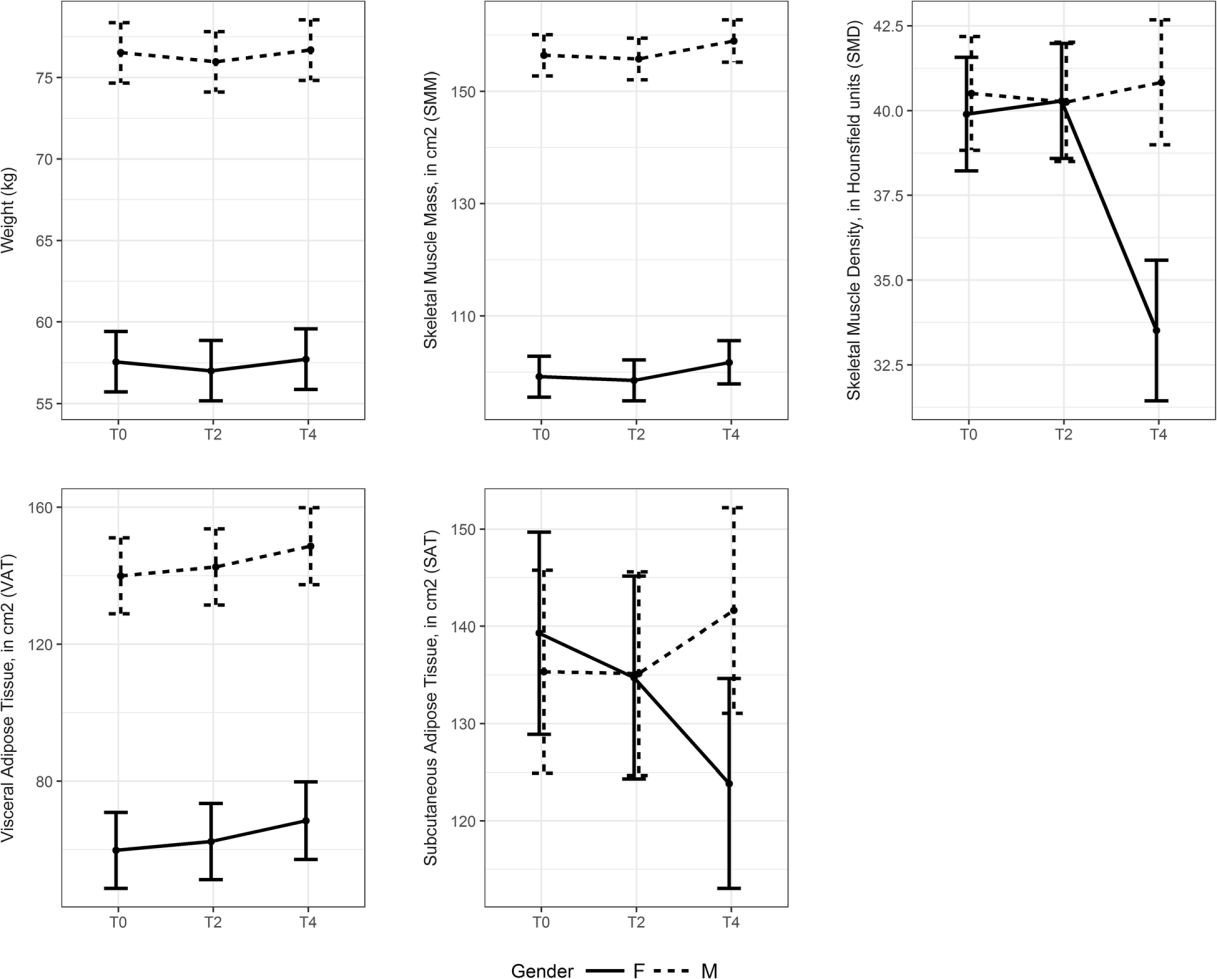
Estimate changes from first CT-scan evaluation. Interaction plot for least square means ± SD for women (solid lines) and men (dotted lines), for weight (kg) between T0-T4 *P* = 0.935, for skeletal muscle mass area (SMM) (cm^2^) between T0-T4 *P* = 0.544, for skeletal muscle density (SMD) (Hounsfield unit) between T0-T4 for women *P* = 0.038 for men *P* = 0.990, for visceral adipose tissue area (cm^2^) between T0-T4 *P* = 0.161, for visceral adipose tissue area (cm^2^) between T0-T4 for women *P* = 0.030 for men *P* = 0.500

Of the 76 patients included in this study, 57 had available CT scans for both the 2 and 4-month follow-up. No decreases were seen in weight, SMM, or VAT during the 4 months of follow-up (Fig. 2, Table 3 and Additional file 1: Table S3). SMD and SAT decreased in women but not men, after 4 months of follow-up, mainly between 2 and 4 months for SMD 6.8 HU (*P* = 0.012), and over the 4-month period for SAT 15.4 cm^2^/m^2^ (*P* = 0.03). The proportion of patients with SMM depletion decreased from 52.4% at baseline to 47.3% after 4 months of treatment.

**Table 3:** Changes in body weight, tissue areas and muscle density during 4 months of follow-up, by gender. Fifty-seven patients were included in body composition changes analysis

	Visit	LS Means [95% CI]	LS Means [95% CI] - Women	LS Means [95% CI] - Men
Weight (kg)	T0	67.04 [64.42; 69.66]	57.57 [53.89. 61.25]	76.51 [72.83. 80.19]
	T2	66.49 [63.86; 69.11]	57.01 [53.33. 60.70]	75.96 [72.28. 79.64]
	T4	67.20 [64.57, 69.84]	57.73 [54.04. 61.42]	76.68 [72.99. 80.37]
SMM (cm^2^)	T0	127.83 [122.47; 133.19]	99.23 [91.96; 106.51]	156.42 [149.14; 163.70]
	T2	127.17 [121.74; 132.59]	98.57 [91.26; 105.89]	155.76 [148.43; 163.10]
	T4	130.36 [124.60; 136.12]	101.76 [94.11; 109.42]	158.95 [151.45; 166.45]
SMD (HU)	T0	40.20 [37.84; 42.56]	39.90 [36.56; 43.23]	40.51 [37.17; 43.85]
	T2	40.27 [37.84; 42.71]	40.29 [36.91; 43.67]	40.26 [36.76; 43.76]
	T4	37.18 [34.41; 39.94]	33.52 [29.38; 37.65]^a^	40.86 [37.16; 44.51]
VAT (cm^2^)	T0	99.86 [83.92; 115.80]	59.77 [37.58; 81.96]	139.95 [117.76; 162.14]
	T2	102.48 [86.48; 118.47]	62.39 [40.16; 84.61]	142.57 [120.33; 164.80]
	T4	108.56 [92.15; 124.96]	68.46 [45.84; 91.09]	148.65 [126.22; 171.07]
SAT (cm^2^)	T0	137.32 [122.65; 152.00]	139.30 [118.57; 160.03]	135.34 [114.57; 156.11]
	T2	134.95 [120.23; 149.67]	134.74 [113.98; 155.51]	135.15 [114.29; 156.01]
	T4	132.75 [117.69; 147.80]	123.85 [102.34; 145.36]^b^	141.64 [120.58; 162.70]

For each body component, the fitted model contained only gender and visit

*LS* lean square means, *CI* Confidence Interval, *SMM* skeletal muscle mass index (cm^2^), *SMD* skeletal muscle density, *HU* Hounsfield Unit, *VAT* visceral adipose tissue (cm^2^), *SAT* subcutaneous adipose tissue (cm^2^)

^a^*P* = 0.038; ^b^*P* = 0.030

**Fig. 3:**
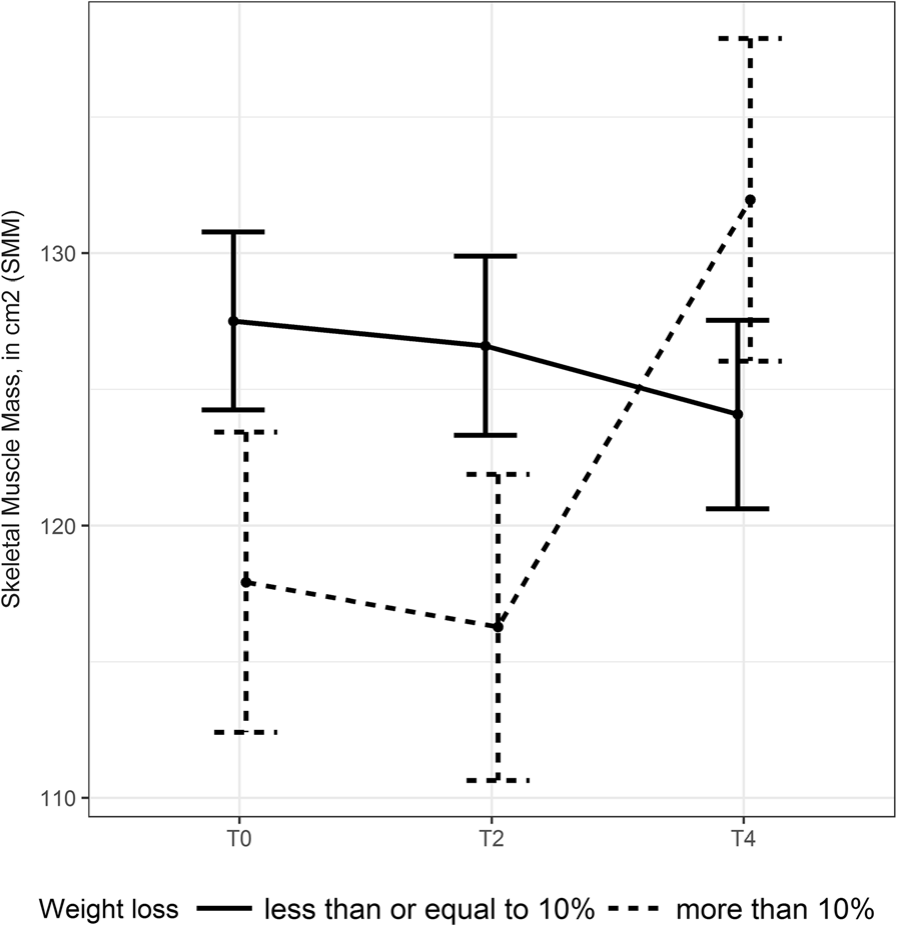
Title: Estimate changes from first CT-scan evaluation for SMM. Legend: Interaction plot for least square means ± SD. Estimate changes from first CT-scan evaluation for SMM between weight loss > 10% (dotted lines) and weight loss ≤10% (solid lines), *P* = 0.008

SMM changes were not influenced by gender, age, initial values of adipose SMM, VAT or SAT, and were not dependent on the presence of SMM loss at baseline (assessed using mixed models). Weight and SMM changes were not influenced by grade 3–4 or any grade of diarrhea. For patients presenting with WL > 10% at baseline, SMM increased significantly after 4 months of follow-up (*P* = 0.008) (Fig. 3).

## Discussion

This study in patients undergoing first-line treatment for mCRC demonstrated a number of noteworthy findings relevant to the role of SMM depletion in cancer patients. The first key observation is that no statistically significant or clinical relevant association was found between low SMM and adverse clinical outcomes. Secondly no changes were observed in weight, SMM, muscle density or adipose tissues during the 4-month follow-up.

Although some studies have described an association between SMM depletion before treatment and OS, we did not observe this association. Analyses in two large CRC cohorts, including 2407 and 804 patients, reported that SMM depletion was an independent prognostic factor for OS [7, 9]. In contrast, McSorely et al. were unable to demonstrate a significant change in OS in CRC patients with SMM depletion [30]. The patients in these three studies presented similar disease characteristics; all had stage I–III CRC, underwent surgical resection and had an abdominal CT scan before surgery. A recent review analyzing contemporary studies concluded that patients with SMM depletion and low SMD are consistently associated with worse survival outcomes [31]. Nonetheless, most of the studies of this systematic review included resectable CRC or cohorts including both respiratory to gastrointestinal cancer patients. The authors also noted that significant methodological heterogeneity, mainly the lack of consensus for cut-offs defining SMM depletion, limited the strength of their conclusions [31]. The only study with a cohort of mCRC similar to ours did not observe an association between SMM depletion at inclusion and OS [18]. However, in contrast to our study, Blauwhoff-Buskerwole et al. observed that SMM loss during follow-up was independently associated with survival (HR 4.47; *P* < 0.001). Differences in anticancer treatments, could potentially explain these discordances. In their study, 22% of the population received a reduced chemotherapy initial dose and only 23% (compared to 88% in our study), received a second-line of chemotherapy, suggesting their population may have had more aggressive disease, or a worse prognosis at inclusion.

It is interesting to note that the first study showing the relationship between SMM depletion and chemotherapy toxicity was reported in CRC patients [32]. This pioneering article was followed by others with similar results [8, 16, 17]. The recent C-SCANS study in a large population (*N* = 533) showed that patients with the lowest tertile of muscle mass were more likely to experience hematologic toxicity and early treatment discontinuation than those in the highest [18]. We however did not identify a relationship between SMM depletion and chemotherapy toxicity but our study sample size did not allow us to adjust for confounding factors. These discrepancies could be linked to our small sample size and/or to different methodology; we analyzed low muscle mass by dichotomous variable (SMM depletion according to Martin’s cut off compared to patients without SMM depletion) rather than with tertiles. The two studies also differ in terms of chemotherapy regimens, with different rates of hematologic toxicity (44% neutropenia in the C-SCANS study compared to 22% here). The above-mentioned studies [8, 16–18] present methodological heterogeneities that limit the possibilities of comparison. The main hurdle is the absence of a defined threshold for SMM depletion and variations in the choice of muscle surfaces used to measure SMM. It would be interesting to repeat the analyses in the C-SCANS study [18] using the cut-off values of SMM depletion used in other studies [6, 9]. This lack of reproducibility could explain why, 10 years after the first publication, SMM is not routinely used for scaling chemotherapy doses.

For women, a decrease in muscle density and SAT was observed after 2 months of follow-up. Decreased SMD could be associated with increased lipid droplet deposit [27]. Stephen et al. carried out a quantitative morphological examination of lipid droplets in muscle biopsies of gastrointestinal cancer patients [33]. Significantly more intramyocellular lipid droplets were seen in patients with lower VAT measures with a trend toward an association between droplet number and SAT. The authors suggest that an increase in lipolysis is responsible for the decreased adipose tissue and an increased deposition of lipids within muscle (low SMD), probably reflecting an imbalance between fatty acid supply and use by muscle. This may explain our results observed in women. Difference in adipose tissue distribution and metabolism according to sex [34] could explain the significant results we observed in women and not in men. We should focus on intrinsic factors such as gender when studying SMM and adipose tissue changes during mCRC treatment and follow-up, as reported by Malietzis et al. [20].

Decreased weight and SMM in cancer patients could be a consequence of reduced dietary intake and/or metabolic changes linked to the cancer or its treatment (49% of patients in our study had lost more than 5% of their usual weight at referral). The most interesting finding is the stabilization of weight and SMM after initiating treatment. Of note, all but one patient in our study had stable disease. This offers supplementary proof that tumor evolution is the main factor impacting weight and SMM loss. Lieffers et al. have reported in mCRC that the most rapid decreases in SMM and adipose tissues were observed when tumor burden increased rapidly [10]. Others have questioned the role of anticancer treatment in SMM and WL. Poterucha et al. observed a decrease in SMM in mCRC patients and suggest that bevacizumab may be responsible [35]. The study by Blauwhoff-Buskerwole et al. showed a 6.1% decrease in SMM in a cohort similar to ours [19]. A recent publication described muscle mass changes during systemic treatment in a comparable, but larger group of 450 mCRC patients [36]. This study found that patients on average lost 0.7 (95% CI -1.11; − 0.26) kg SMM during the 4 months of first line intensive (carboplatine-oxaliplatine-bevacizumab treatment CAPOX-B). Subsequently SMM recovered after subsequent less intensive carboplatine-oxaliplatine or observation treatment, and again decreased during more intensive CAPOX-B or other reintroduction treatment [36]. From these two studies with the only apparent difference between our population and theirs being the chemotherapy regimens (irinotecan versus oxaliplatin respectively), we can hypothesize that the SMM decrease observed in the Blauwhoff-Buskerwole and Kurk studies is a direct effect of oxaliplatine, as described for other anti-cancer regiment: targeted therapies [22], doxorubicin [37, 38], and cisplatin [39]. This is not however supported by a study in mice showing that unlike oxaliplatin, irinotecan causes marked depletion in SMM [40]. Together, these findings highlight the importance of considering the toxic effects of chemotherapy on SMM, and could explain the observed discordant results.

In addition to metabolic abnormalities, another component relating to WL in cancer patients is decreased food intake. In 1131 hospitalized CRC patients, unintentional WL was associated with decreased intake [41]. In our population, all grade diarrheas was observed in 74% of cases, with grade 3–4 in 14%. Despite these digestive and nutritional symptoms, which can decrease nutriment intake, we did not observe any consequences on weight or SMM, supporting the absence of nutritional disorders during early mCRC treatment despite gastrointestinal symptoms linked to chemotherapy toxicities.

WL and SMM depletion in mCRC patients during first-line anticancer chemotherapy seem not to be influenced by the initial nutritional status since no association was seen between the rate of SMM loss and WL for any nutritional parameters at inclusion, including BMI, SMM, muscle density, SMM depletion and adipose tissue. The only parameter associated with the rate of SMM loss is WL prior to inclusion, with significantly increased SMM during 4 months of follow-up in patients with WL > 10% at inclusion. We can hypothesize that SMM could be restored by treatments which control cancer evolution and that worse the metabolic abnormality causing muscle protein breakdown the greater this effect, reaching a critical level of WL > 10%.

We are aware of several limitations of this study. Our study was designed to assess if previous findings about body composition are reproducible for different anticancer therapies; however, due to its small sample size and since consecutive CT images were available in only part of the patients, our study was exploratory exclusively. Of note, this small sample size induces a low power and prevented us from adjusting toxicity analyses for confounding factors. Another limitation is the delay between patient’s inclusion and our study. It may be considered a long one not representing the current management and the current treatment of CRC in the time of molecular abnormality screening and targeted therapies.

## Conclusion

In a homogenous cohort of mCRC patients treated with first-line chemotherapy, SMM depletion was not significantly associated with adverse clinical outcomes in terms of either survival or chemotherapy toxicities. These results differ from those observed by other groups, clearly highlighting the need for standardized definitions of SMM depletion thresholds and of the muscle surfaces to measure SMM. Limiting methodological heterogeneity will improve our understanding of the prognostic role of SMM depletion. The fact that we did not observe any changes in weight and SMM during the first 4 months of follow-up is surprising given the hypercatabolism observed in mCRC (half of the patients had WL before treatment), the high incidence of gastrointestinal toxicity, and the potential protein breakdown effect of chemotherapy. Our study also provides supplementary proof that tumor evolution is the main factor of weight and SMM loss, with stable disease, irrespective of nutritional and gastrointestinal disorders, maintaining weight and SMM. This highlights the important role of metabolic alterations and systemic inflammatory processes in muscle wasting. However, our results are not based on a large sample size, they did not indicate that SMM depletion was associated with adverse clinical outcomes, and further studies are necessary before drawing definitive conclusions.

## Additional file

Additional file 1: **Table S1.** Univariate and multivariable analyses for progression-free survival. **Table S2.** Chemotherapy toxicities on treatment according to Skeletal Muscle Mass loss. **Table S3**. Rates of change in weight, tissue areas, and muscle density during 4 months of follow-up. (DOCX 48 kb)

## Data Availability

The datasets used and/or analyzed in the current study are available from the corresponding author on reasonable request.

## Abbreviations

BC: Body composition
BMI: Body mass index
CT: Computed tomography
mCRC: Metastatic colorectal cancer
OS: Overall survival
SAT: Subcutaneous adipose tissue
SMD: Skeletal muscle radiodensity
SMM: Skeletal muscle mass
VAT: Visceral adipose tissue
WL: Weight loss
