# Supplementary material for: No evidence for changes in skeletal muscle mass or weight during first-line chemotherapy for metastatic colorectal cancer
Source: BMC Cancer. 2019 Aug 28;19:847. doi: 10.1186/s12885-019-6086-2 (PMC6714393; doi:10.1186/s12885-019-6086-2)
Supplement: Supplementary file 1 — Table S1. Univariate and multivariable analyses for progression-free survival. Table S2. Chemotherapy toxicities on treatment according to Skeletal Muscle Mass loss. Table S3. Rates of change in weight, tissue areas, and muscle density during 4 months of follow-up. (DOCX 48 kb) [file 12885_2019_6086_MOESM1_ESM.docx]

# Table S1:

# Univariate and multivariable analyses for progression-free survival

|  |  |  | Univariate analysis | | Multivariable analysis | |
| --- | --- | --- | --- | --- | --- | --- |
| Characteristic | No. evt/ No. pts | Median PFS (95% CI) | HR [95% CI] | *P*-value | HR [95% CI] | *P*-value |
| Age (years) |  |  | 1.03 [1.00 ; 1.06] | 0.020 | 1.04 [1.00 ; 1.08] | 0.038 |
| Sex |  |  |  |  |  |  |
| Male | 29/37 | 9 (8 ; 14) | 1 | 0.310 |  |  |
| Female | 34/38 | 10 (9 ; 11) | 1.30 [0.78 ; 2.17] |  |  |  |
| ECOG performance status |  |  |  |  |  |  |
| 0-1 | 56/68 | 9 (9 ; 11) | 1 | 0.180 |  |  |
| 2 | 7/7 | 9 (4 ; 10) | 1.72 [0.77 ; 3.83] |  |  |  |
| Number of metastatic sites |  |  |  |  |  |  |
| 1 | 22/28 | 10.5 (9 ; 14) | 1 | 0.066 | 1 | 0.013 |
| ≥2 | 41/47 | 9 (8 ; 10) | 1.64 [0.97 ; 2.77] |  | 1.97 [1.15 ; 3.38] |  |
| Treatment |  |  |  |  |  |  |
| XELIRI | 23/31 | 10 (9 ; 15) | 1 | 0.055 | 1 | 0.020 |
| FOLFIRI | 40/44 | 9 (8 ; 10) | 1.67 [0.99 ; 2.8] |  | 1.90 [1.11 ; 3.26] |  |
| Body mass index category (kg/m2) |  |  |  |  |  |  |
| <25 | 38/45 | 9 (9 ; 10) | 1 | 0.820 |  |  |
| ≥25 | 25/30 | 10 (8 ; 14) | 0.94 [0.57 ; 1.57] |  |  |  |
| SMM loss |  |  |  |  |  |  |
| No | 30/35 | 9 (8 ; 11) | 1 | 0.840 |  |  |
| Yes | 33/40 | 9 (9 ; 10) | 0.95 [0.58 ; 1.56] |  |  |  |
| L3 skeletal muscle score |  |  | 0.99 [0.77 ; 1.26] | 0.930 |  |  |
| L3 visceral adipose tissue score |  |  | 1.12 [0.88 ; 1.43] | 0.340 |  |  |
| L3 subcutaneous adipose tissue score |  |  | 0.90 [0.69 ; 1.18] | 0.450 |  |  |
| L3 total adipose tissue score |  |  | 1.03 [0.79 ; 1.34] | 0.830 |  |  |
| Skeletal muscle density (HU) |  |  | 0.97 [0.95 ; 1.00] | 0.048 | 1.00 [0.96 ; 1.04] | 0.970 |
| No. evt: number events; No. pts: number patients; HR: Hazard Ratio; CI: confidence interval; p-value; ECOG Eastern Cooperative Oncology Group; HU: Hounsfield Units  Skeletal Muscle mass (SMM) loss thresholds were those defined by Martin et al. [6 | | | | | | |

# Table S2:

# Chemotherapy toxicities according to Skeletal Muscle Mass loss

|  | Without SMM loss n=36 | With SMM loss n=40 | Overall  n=76 | *P*-value |
| --- | --- | --- | --- | --- |
| During 2 months of treatment |  |  |  |  |
| Diarrhea 3-4 | 1 (3%) | 7 (18%) | 8 (11%) | 0.059 |
| Diarrhea 1-2 | 21 (58%) | 21 (52%) | 42 (55%) | 0.780 |
| Neutropenia 3-4 | 6 (17%) | 6 (15%) | 12 (16%) | 1 |
| Neutropenia 1-2 | 15 (42%) | 20 (50%) | 35 (46%) | 0.619 |
| Hypertension or proteinuria | 8 (22%) | 10 (25%) | 18 (24%) | 0.989 |
| Any grade 3-4 AE | 13 (36%) | 21 (52%) | 34 (45%) | 0.229 |
| During 4 months of treatment |  |  |  |  |
| Diarrhea 3-4 | 4 (11%) | 7 (18%) | 11 (14%) | 0.643 |
| Diarrhea 1-2 | 30 (83%) | 26 (65%) | 56 (74%) | 0.121 |
| Neutropenia 3-4 | 8 (22%) | 9 (22%) | 17 (22%) | 1 |
| Neutropenia 1-2 | 18 (50%) | 26 (65%) | 44 (58%) | 0.276 |
| Hypertension or proteinuria | 12 (33%) | 15 (38%) | 27 (36%) | 0.889 |
| Any grade 3-4 AE | 20 (56%) | 27 (68%) | 47 (62%) | 0.404 |
| Dose-limiting toxicity causing: |  |  |  |  |
| Dose reduction | 14 (39%) | 24 (60%) | 38 (50%) | 0.108 |
| Treatment discontinuation | 19 (53%) | 20 (50%) | 39 (51%) | 0.990 |
| Treatment discontinuation or reduction | 21 (58%) | 27 (68%) | 48 (63%) | 0.556 |
| Data are presented as the number and percentage of patients, NCI-CTCAE v3.0  Skeletal Muscle mass (SMM) loss thresholds were those defined by Martin et al. [6] | | | | |

# Table S3:

# Rates of change in weight, tissue areas, and muscle density during 4 months of follow-up. 57 Patients were included in body composition changes analysis

| Absolute changes per week | | Estimate | *P*-value | Estimate - Women | *P*-value – Women | Estimate - Men | *P*-value - Men |
| --- | --- | --- | --- | --- | --- | --- | --- |
| Estimated slope of weight (kg/week) | | | |  |  |  |  |
|  | T0 - T2 | 0.55 | 0.222 |  |  |  |  |
|  | T0 - T4 | -0.16 | 0.935 |  |  |  |  |
|  | T2 - T4 | -0.72 | 0.096 |  |  |  |  |
| Estimated slope of SMM (cm^2^/week) | | | |  |  |  |  |
|  | T0 - T2 | 0.66 | 0.935 |  |  |  |  |
|  | T0 - T4 | -2.53 | 0.544 |  |  |  |  |
|  | T2 - T4 | -3.19 | 0.302 |  |  |  |  |
| Estimated slope of SMD (HU/week) | | | |  |  |  |  |
|  | T0 - T2 | -0.07 | 0.999 | -0.39 | 0.979 | 0.25 | 0.992 |
|  | T0 - T4 | 3.03 | 0.200 | 6.38 | 0.038 | -0.33 | 0.990 |
|  | T2 - T4 | 3.10 | 0.134 | 6.77 | 0.012 | -0.58 | 0.962 |
| Estimated slope of VAT (cm^2^/week) | | | |  |  |  |  |
|  | T0 - T2 | -2.62 | 0.690 |  |  |  |  |
|  | T0 - T4 | -8.70 | 0.161 |  |  |  |  |
|  | T2 - T4 | -6.08 | 0.229 |  |  |  |  |
| Estimated slope of SAT (cm^2^/week) | | | |  |  |  |  |
|  | T0 - T2 | 2.38 | 0.668 | 4.55 | 0.462 | 0.20 | 0.999 |
|  | T0 - T4 | 4.57 | 0.508 | 15.45 | 0.030 | -6.31 | 0.500 |
|  | T2 - T4 | 2.19 | 0.773 | 10.90 | 0.059 | -6.51 | 0.292 |
| Tissues area measured in cm^2^;  SMM: skeletal muscle mass index (cm^2^); SMD: skeletal muscle density; HU: Hounsfield Unit; VAT: visceral adipose tissue (cm^2^); SAT: sub cutaneous adipose tissue (cm^2^) | | | | | | | |
